# Sitting Tight While Others Fight

**Published:** 1919-05-17

**Authors:** 


### SITTING TIGHT WHILE OTHERS FIGHT.

The barometer of the Central Committee points to storm, and all the meteorological conditions indicate disturbance. In The Hospital of April 26 I challenged the Central Committee to establish their claim to a membership of 30,000 through which they have so far succeeded in securing a highly inflated representation on the Registration Council proposed to be established by the Bill now in Parliament. I instanced two Irish associations, which I stated are in reality one and the same so far as membership is concerned, and pointed out that these cannot be regarded as representative of Irish nurses. The Central Committee, voiced by Mrs. Fenwick in the *British Journal of Nursing*, declines the challenge in characteristic fashion, as I anticipated. "Our policy," remarks Mrs. Fenwick, "is to sit tight!" But she refers the battle, if battle there is to be, to Ireland. Let the Irish do the fighting.

What happened was this. As soon as Mrs. Fenwick read her Hospital she immediately sat down and posted a copy to Miss Carson Rae, who proceeded straightway to summon the I.N.A. and the I.N.B. to conference. They met accordingly on the succeeding Friday and Saturday to discuss the situation, and at each it was unanimously decided to seek Counsel's opinion as to whether an action lies for libel. The word "bogus," as alleged to be applied to these Societies, is regarded by those concerned as coming within the long arm of the law. As I am neither Counsel nor Jury I do not know.

However, I may point out that I did not suggest that either Society is in its entirety bogus. I am aware that they are not, and Mrs. Fenwick's Editorial in the issue of her Journal of 3rd inst is lacking in accuracy. She has taken the jewel from its setting. What I said then I now repeat, that "as being institutions representative of Irish nurses, both the I.N.A. and the I.N.B. are bogus, absolutely and utterly." To remove the condition is to turn truth into falsehood. It is presumably as representative institutions that they are placed on the Registration Council. Now as to the use of the word "bogus," whether it is justifiable or not, I leave the reader to form an unbiased opinion.

The Hard Facts.

It is estimated by those most competent to form an opinion that the nursing strength of Ireland is 7,000. It is impossible to verify the figure, but this is the common belief. The Irish Nurses Association admits four classes: (*a*) Trained members. (*b*) Professional associates. (*c*) Probationer associates. (*d*) Hon. members. Professional associates include certified midwives, masseuses, and mental nurses. The annual subscriptions are three shillings for members and half-a-crown for associates. During the year 1918 the number who paid subscriptions to this quadruple alliance was less than four hundred. Is it justifiable or otherwise to regard this Association as representative of a 7,000 electorate?

The Irish Nursing Board, which is an institution of yesterday, is in its constitution thoroughly sound. Its President is Colonel Sir Arthur Chance. Some weeks ago I had occasion to refer to Sir Arthur Chance in The Hospital in connection with the meeting held as a consequence of the forming of an Irish Trades Union for Nurses. I pointed to him as one of Dublin's foremost surgeons and most popular figures, the ardent friend and advocate of unity, and the uncompromising opponent of faction. I recall this, and repeat it. The Constitution of the Board is carefully drawn and is above reproach. But when all this is said the fact remains that its total membership does not exceed 400. The members consist of "all persons who can satisfy the Board of their competence as nurses." Moreover, the greater of these institutions, being the I.N.B., includes the less. In other words a massed band of the trained nurses included in both societies would not exceed the membership of the Irish Nursing Board, the members of which have each paid a guinea subscription, which is to entitle them to enrolment on the State register.

To my mind, that these two Associations, being one and the same to all intents and purposes, but having a dual function, should claim in the first place to represent 7,000 nurses, and in the second to send two representatives to the Council of the United Kingdom is monstrous. Metropolitan Hospitals could in many instances on the same basis claim to put forward a representative. Why, may I inquire, should not the newly-formed Irish Trades Union of Nurses send forward a representative? Its membership, I am informed, exceeds four hundred, although it is in its babyhood, and Irish nurses are at least as interested in its success as in that of either the I.N.A. or the I.N.B.

Mrs. Fenwick describes my "attack" as "cowardly and untrue." Can she controvert the facts as herein set out? I give figures, and the figures constitute the argument. It is not a matter of words, nor of dignity, nor of former good works, real or imaginary. It is a case of proportional representation. Egging on another Irish fight from 431 Oxford Street, and hugging the policy of "sitting tight" is slim. But it is not statesmanship. IERNE.

